# Evolution of public beliefs about schizophrenia and attitudes towards those afflicted in Austria over two decades

**DOI:** 10.1007/s00127-020-01963-0

**Published:** 2020-10-30

**Authors:** Matthias C. Angermeyer, Alfred Grausgruber, Elisabeth Hackl, Robert Moosbrugger, Dimitri Prandner

**Affiliations:** 1 Center for Public Mental Health, Untere Zeile 13, 3482 Gösing am Wagram, Austria; 2 Department for Sociology - Empirical Social Research Unit, Johannes Kepler University Linz, Altenbergerstraße 69, 4040 Linz, Austria; 3 Department for Political Science and Sociology, Paris Lodron University Salzburg, Kapitelgasse 4/6, 5020 Salzburg, Austria

**Keywords:** Attitudes, Beliefs, Schizophrenia, Austria, Time trend

## Abstract

**Purpose:**

In the recent years, it was possible to observe two trends: First, there has been a trend to greater mental health literacy, in particular towards a biological model of schizophrenia. Secondly, an increase in public acceptance of professional help and psychiatric treatment has been observed in western countries. This indicates that the societal idea about mental illness and how it can be treated has changed. However, no changes or even changes to the worse occurred regarding the attitudes towards those suffering from the illness, particularly concerning schizophrenia. Thus, the question arises as to whether similar trends can also be found in Austria.

**Methods:**

We use data from two representative population surveys in Austria, conducted 1998 (*n* = 1042) and 2018 (*n* = 1010) using face-to-face interviews, the same sampling procedure, interview mode, and interview schedule.

**Results:**

The data show that today Austrians tend to opt less frequently for genetic factors and chronic stress as causes of schizophrenia than 20 years ago. There were only slight changes regarding intended first help-seeking actions except for a stronger endorsement of lay help. The believe in an effective treatment of schizophrenia has increased significantly and there was a marked trend towards preference of medication over psychotherapy. Social acceptance of people with schizophrenia has increased, but also the ascription of violence.

**Conclusion:**

In summary, the evolution of attitudes and beliefs concerning schizophrenia in Austria shows a rather inconsistent pattern and differs to some extent from what has been observed in other western countries. This is important to know when planning awareness-raising or stigma-reducing initiatives.

## Introduction

In the recent years, studies examining time trends of public attitudes and beliefs about mental disorders in general, and specifically schizophrenia, have been carried out in several western countries. Those captured several different trends, which were summarized in a meta-analysis of vignette-based trend studies [1]. The identified studies featured national representative population samples, were conducted until 2007 and covered 11 years at the most. On a content level the meta-analysis revealed a coherent drift towards greater mental health literacy. In particular, a trend towards a biological model of schizophrenia, and a significant increase in public acceptance of medication for the treatment of the disorder could be reported. In contrast, no change was found in the ascription of dangerousness to people with schizophrenia, the desire for social distance had even increased [1].

In the meantime, three trend studies, whose previous waves were covered in the afore mentioned meta-analysis, have seen further surveys, adding new assessments concerning public attitudes. In Germany, a third survey dealing with such issues was conducted in 2011, building on surveys completed in 1990 and 2001, prolonging the observation period to 21 years [2]. Confirming the already reported findings of the meta-analysis [1], it illustrates that during this time period the public’s inclination to endorse a biological causation of schizophrenia and its readiness to recommend help-seeking from mental health professionals as well as using psychotropic medication and psychotherapy has increased considerably [2]. Furthermore, this study also came to the conclusion that the emotional reactions to people with schizophrenia have worsened and the desire for social distance has increased [2].

The second trend study originates from Australia, where, in addition to surveys in 1995 and 2003/4, a third survey was conducted in 2011. Over a 16-years period, belief in inherited or genetic causes of schizophrenia had increased [3]. The results also showed a growing belief in the helpfulness of mental health professionals and psychotropic medication for the treatment of schizophrenia. Beliefs about effective medications and interventions moved closer to those of mental health professionals [4]. Thus, between 2003/4 and 2011 (there is no data available for 1995) the perception of people with schizophrenia being dangerous increased significantly, while respondents’ unwillingness to socially interact with such a person did not change significantly [5].

In the US, in addition to previous surveys in 1996 and 2006, a third population survey was conducted in 2018. So far only the results on the ascription of violence to people with mental illness have been published, showing that over a 22-years period the percentage of the American public seeing people with schizophrenia as dangerous to others rose significantly [6].

In the present paper, we report the results from a fourth study investigating long-term trends in attitudes and beliefs about schizophrenia. The study has been carried out in Austria. The first survey was conducted in 1998 [7], the second in 2018, resulting in an observation period of 20 years. Using data from this study, we will examine whether the trends observed in previous studies in other countries can also be found in Austria.

More specifically, we will address the following questions:

1. How have the Austrian public’s beliefs about the causes of schizophrenia developed over the past twenty years? Has there also been a growing tendency to attribute the etiology of the illness to biogenetic factors?
2. Does in 2018 the public show the same help-seeking reactions in case of a relative’s or acquaintance’s illness as twenty years before, or are there some differences? Particularly, has in Austria, as in other countries, the readiness to seek help from mental health professionals increased?
3. In which way have the Austrian public’s beliefs about the treatment of schizophrenia changed over the past two decades? Has there also been a growing acceptance of psychopharmacological medication?
4. How have public attitudes towards people with schizophrenia (e.g., desire for social distance, perceived dangerousness, and attitude towards integration into society) developed in Austria since 1998?

## Methods

### Surveys, interviewing, and analysis

The analyses presented in this article are based on data from two population-based trend surveys among Austrian citizens. The first survey was conducted in 1998 (*n* = 1042), the second in 2018 (*n* = 1010). In 2018, the sampling frame was based on the address register of the Austrian postal service, targeting individuals aged 16 years and older. A multistage random sampling has been used with a response rate of 48%. In 1998, quota samples have been drawn.

The sociodemographic characteristics for both samples and, for comparison, those of the general population in the respective years are reported in Table 1. Since there were no statistically significant differences, the samples can be considered representative of the Austrian population in the corresponding years. A combination of design and poststratification weights was applied for descriptive purposes. The poststratification weights were calculated based on the population level distributions of educational achievements, age, and sex. The reference for each year was the Austrian micro census, a mandatory large-scale population survey tracking the national social structure.

**Table 1:** Sociodemographic characteristics of both population samples

	Sample 1998^a,^^e^	General population 1998^b,^^e^	Sample 2018^c,^^e^	General population 2016^d,^^e^
Gender (%)				
Male	48.1	48.0	48.7	48.9
Female	51.8	52.1	51.3	51.2
Age, years (%)				
16–29	22.9	23.0	18.7	20.5
30–39	21.3	21.2	16.0	15.9
40–49	16.5	16.5	18.0	17.6
50–59	15.2	15.1	18.2	18.1
60–69	10.9	10.9	13.0	12.9
≥ 70	13.1	13.4	16.0	15.1
Educational level ≥ Matura^f^ (%)	20.7	19.6	28.0	28.8

^a^Results from the baseline study in 1998, weighted data

^b^Results from the Austrian microcensus 1998

^c^Results from the MOPUSTIA18, weighted data

^d^Results from the Austrian microcensus 2016

^e^Because of rounding figures will not always show equal differences

^f^Matura indicates that someone completed secondary education (at least 12 years of schooling)

Fieldwork was carried out in 1998 by the *Institut für Markt- und Sozialanalysen Ges.m.b.H. (IMAS; Linz)* and in 2018 by the *Institut für empirische Sozialforschung GmbH (IFES; Vienna).* Both companies are specialized in market and social research. Informed consent was considered to have been given when individuals accepted to complete the interview.

*Data collection* for both presented surveys was conducted via face-to-face interviews, administrated by trained interviewers. In 1998 pen and paper interviews were conducted, in 2018 computer-assisted personal interviews. In accordance with the recent methodological literature on surveys as well as a case study from Austria there is no indication, that this may have introduced a substantial mode effect [8, 9]. The interview sections used for this analysis were identical in wording, answer categories and the sequence of questions. After having elicited the knowledge regarding and associations with the term ‘schizophrenia’, respondents were presented with a short description (vignette) of the psychopathology of schizophrenia plus a brief depiction of a person suffering from the disorder. Then, respondents were asked a series of questions to assess their attitudes towards people with schizophrenia as well as their causal and treatment beliefs and their potential help-seeking actions in case of a relative’s/acquaintance’s illness.

### Measurements

The first central domain discussed refers to *beliefs about possible causes* of schizophrenia. Respondents were confronted with a list of potential causes, comprising social–environmental factors (bad family background, bad housing conditions), stress-related factors (nervous overstrain, stress at work, and negative life events), genetic factors, and personal factors (lack of willpower, excessive lifestyle). Using a four-point Likert scale, respondents should indicate for each cause how often they believed it be responsible for the etiology of schizophrenia (very frequently – rather frequently – rather rarely – never; do not know).

In order to assess respondents’ *help-seeking intentions* they were then asked what they would do first when someone among their relatives or acquaintances would suddenly suffer from such an illness. They could choose between the following options: try to talk to him/her and to solve the problem within the family; ask acquaintances or neighbors for advice; ask a teacher or a pastor or another person competent and experienced in such matters for advice; read a book about this condition or consult the internet; turn to a G.P.; go to a specialist (psychiatrist or neurologist); see a psychologist/psychotherapist; arrange for the admission to a psychiatric hospital or a psychiatric ward in a general hospital; do not know.

*Appraisal of the effectiveness of treatment* was assessed asking ‘Do you think that there exists an effective treatment for someone with such an illness?’ (response categories yes, no, do not know). Respondents believing in the effectiveness of treatment were then invited to select one of the following options: Medication only; medication as first-line treatment, but also psychotherapy or counseling; psychotherapy or counseling as first-line treatment, but also medication; psychotherapy or counseling only; other; do not know.

*Social acceptance* of persons with schizophrenia was assessed by means of the following five questions, based on the social distance scale developed by Link et al. [10]: ‘Would you trust such a person to watch over your children?’; ‘Supposed you were employer: Would you hire such a person?’; ‘Would you accept having such a person as your superior?’ ‘Would you agree with such a person to marry into your family?’; ‘Would you feel comfortable having such a person as a neighbor?’ Respondents could indicate whether they would, or would not accept, in the situations presented the person described in the vignette. In addition, ‘do not know’ options were provided.

Respondents’ *ascription of violence to people with schizophrenia* was assessed asking ‘What about people suffering from schizophrenia: Do you believe they tend to be more violent than people without mental health problems, or not?’ Response categories were ‘Yes, I rather tend to believe that’, ‘No. I do not tend to this opinion’ and ‘I do not know’.

To assess respondents’ *attitude towards integrating people with schizophrenia into society*, they were questioned: ‘Do you think that someone with such an illness should live in our midst?)’. Respondents could answer with ‘Yes’, ‘No’ or ‘I do not know’.

Statistical analyses presented in this article were done with *IBM SPSS V26*. As stated, the data was weighted for representation purposes; with the lowest weight applied being 0.3 multiplier, and the highest a 3.6 multiplier. This helped to establish representation concerning the descriptive statistics. There were no indications that the applied weights influenced the multivariate analysis substantially. The analytical procedures done for the study were based on logistic regression modeling to identify significant influences on the public attitudes concerning schizophrenia, while controlling for age, sex and formal education. Comparisons between the surveys were based on mean-value comparisons as well as an ANOVA. The model specifications are noted in the results section of the article.

## Results

### Causal attributions

As shown in Table 2, across the two decades, stress-related and genetic factors were considered as prevailing causes for schizophrenia. However, except for negative life events, in 2018 respondents tended to opt significantly less for them than 20 years ago. In comparison, over time, rather little importance was ascribed to social–environmental and personal factors. Still, the probability that bad housing conditions as well as a personal excessive lifestyle are seen as causes of schizophrenia has increased significantly.

**Table 2:** Changes in the public’s beliefs about the causes of schizophrenia between 1998 and 2018

Response category: a cause	Predicted percentages			*p*^b^
	1998	2018	Change (95% CI)^a^	
Bad family background	44	45	0 (− 4.5)	0.787
Bad housing conditions	17	25	**8 (5.12)**	**0.001**
Negative life events	73	69	− 4 (0.8)	0.096
Nervous overstrain	72	65	− **7 (3.11)**	**0.001**
Occupational stress	57	51	− **6 (2.10)**	**0.010**
Heredity	76	70	− **6 (2.10)**	**0.003**
Weak will	31	28	− 3 (1.7)	0.428
Excessive lifestyle	20	29	**9 (5.13)**	**0.001**

Results from ANOVA and logistic regressions, under statistical control of age, gender, educational level, data are weighed dichotomized variables: very often + often/seldom + never

^a^Because of rounding figures will not always show equal differences; figures in parentheses show values of confidence interval (lowest and highest values)

^b^Statistically significant changes are in bold, *p* values are the results of logistic regression analyses, under statistical control of gender, age, educational level

### Help-seeking intentions

The study shows that over the last 20 years, there are only slight differences regarding intended first help-seeking actions in the case that either a relative or acquaintance is suffering from schizophrenia (see Table 3). The ranking generally remains the same: The consultation of specialists (psychiatrist, neurologist) is still the most opted for first help-seeking action, followed by the attempt to talk to the concerned person or to solve the problem within the family, which both are more frequently endorsed in 2018. On the contrary, turning to a general practitioner is less frequently proposed, but still ranks third. Asking acquaintances or neighbors for advice, obviously is now more frequently taken into consideration than in the past. In 2018, it equals seeing a psychologist/psychotherapist as possible first source of help. Reading a book or consulting the Internet, seeking advice from competent/experienced people as well as arranging the admission to a psychiatric hospital are still options chosen from only a small minority.

**Table 3:** Changes in help-seeking intentions between 1998 and 2018

Response category: would do firstly	Predicted percentages			*p*^b^
	1998	2018	Change (95% CI)^a^	
Try to talk with him/her and solve the problem within the family	24	29	**5 (1.9)**	**0.005**
Ask acquaintances or neighbors for advice	4	8	**5 (3.7)**	**0.000**
Ask a teacher or a pastor or another person competent and experienced in such matters for advice	2	4	2 (1.3)	0.099
Read a book about this condition or consult the internet	6	5	− 1 (− 1.3)	0.392
Turn to a general practitioner	17	12	− **5 (2.8)**	**0.002**
Go to a specialist (psychiatrist or neurologist)	32	30	− 2 (− 2.6)	0.133
See a psychologist/ psychotherapist	11	9	− 2 (0.4)	0.238
Arrange for the admission to a psychiatric hospital or a psychiatric ward in a general hospital	5	3	− 1 (0.3)	0.159

Results from ANOVA and logistic regressions, under statistical control of age, gender, educational level, data are weighed, dichotomized variables: yes/no

^a^Because of rounding figures will not always show equal differences; figures in parentheses show values of confidence interval (lowest and highest values)

^b^Statistically significant changes are in bold, *p* values are the results of logistic regression analyses, under statistical control of gender, age, and educational level

### Treatment beliefs

The probability of the public to believe in the treatability of schizophrenia increased significantly over the last two decades: While in 1998 56% of those questioned shared the view that there exists an effective treatment for schizophrenia, in 2018 the proportion had increased to 75% (change + 19; 95% CI 15.23; *p* = 0.000). Moreover, among those in favor of treatment, a marked trend towards preference of medication over psychotherapy has emerged (see Table 4). Whereas in 1998 psychotherapy, accompanied by medication, was clearly (60%) considered as first-line treatment for people with schizophrenia, nowadays “only” 31% endorse this treatment option. In contrast, in 2018 more than half of the respondents believed that medication, supported by psychotherapy, is the most primary effective treatment option; nowadays, 15% even plead for medication alone.

**Table 4:** Changes in beliefs about the effectiveness of medication and psychotherapy/counseling for the treatment of schizophrenia between 1998 and 2018

Response category: effective treatment	Predicted percentages			*p*^b^
	1998	2018	Change (95% CI)^a^	
Medication only	8	15	**7 (3.11)**	**0.000**
Medication as first-line treatment	27	51	**24 (19.29)**	**0.000**
Psychotherapy or counseling as first-line treatment	60	31	− **29 (24.35)**	**0.000**
Psychotherapy or counseling only	5	3	− 2 (0.4)	0.138

Results from ANOVA and logistic regressions, under statistical control of age, gender, educational level, data are weighed, dichotomized variables: yes/no

^a^Because of rounding figures will not always show equal differences; figures in parentheses show values of confidence interval (lowest and highest values)

^b^Statistically significant changes are in bold, *p* values are the results of logistic regression analyses, under statistical control of gender, age, and educational level

### Attitudes towards persons with schizophrenia

Over the past 20 years, social acceptance of people with schizophrenia has increased considerably. As shown in Table 5, except for having such a person as neighbor, across all social situations the Austrian public has become more willing to engage in social relationships with someone with schizophrenia.

**Table 5:** Changes in social acceptance of people with schizophrenia between 1998 and 2018

Response category: would accept	Predicted percentages			*p*^b^
	1998	2018	Change (95% CI)^a^	
Take care of children	11	14	**3 (0.6)**	**0.040**
Hire as employee	36	41	**5 (1.9)**	**0.029**
Accept as superior	19	41	**21 (17.25)**	**0.001**
Marriage into family	32	45	**13 (9.18)**	**0.001**
Accept as neighbour	72	75	3 (0.7)	0.147

Results from ANOVA and logistic regressions, under statistical control of age, gender, educational level, data are weighed, dichotomized variables: yes/no

^a^Because of rounding figures will not always show equal differences; figures in parentheses show values of confidence interval (lowest and highest values)

^b^Statistically significant changes are in bold, *p* values are the results of logistic regression analyses, under statistical control of gender, age, educational level

The public’s attitude towards the integration of people with schizophrenia into society remained pretty much the same across the last two decades. No significant changes are observable in this regard: Did in 1998 81% of respondents accept that people with schizophrenia live in their midst, in 2018 the proportion amounted to 84% (change + 2; 95% CI 0.6; *p* = 0.198).

In contrast to the growing social acceptance, the probability that people with schizophrenia were perceived as violent also increased significantly over the observation period, from 55% in 1998 to 60% in 2018 (change + 5; 95% CI 1.10; *p* = 0.012).

## Discussion

Main aim of our study was to examine whether trends in public attitudes and beliefs about schizophrenia, that have been observed in previous studies, can be replicated in Austria.

As mentioned in the introduction, parallel to developments within psychiatry, a growing popularity of biogenetic conceptualizations of schizophrenia could be observed among the public in other western countries [1–3, 6]. Regarding this trend, it has to be stated, that this seems not to be the case for Austria, where genetic factors were less frequently endorsed in 2018 than 20 years ago.

In addition, causal factors not approved by mental health experts such as bad housing conditions or excessive personal lifestyle enjoyed in 2018 even greater approval than in 1998. Thus, in Austria the gap between conceptualizations held by the general public and by mental health professionals, has not narrowed over the past 20 years. There are signs that the opposite may be true. However, the currently available data material does not provide any satisfying answers, how such developments in Austria can be explained. Furthermore, the existing literature does not offer comparable studies that may provide additional insights.

When it comes to help-seeking intentions and treatment beliefs, the results are mixed. During the two decades under study, trust in the effectiveness of treatment has increased, with 65.9% (*n* = 621) of the surveyed individuals in 2018 favoring medication to treat schizophrenia. At the same time psychotherapy has lost ground and was more frequently considered as an adjunct to medication, among those who think that schizophrenia is treatable.

So far, our findings concur with those of other trend studies [1]. However, and in contrast to previous studies, the Austrian public’s readiness to seek help from mental health professionals as first reaction if someone else is suffering from schizophrenia has not increased. Instead, lay help (i.e., help provided by family, friends and acquaintances) has become more popular as first source of help. Nevertheless 30% of the respondents answered that seeking professional help is very important. Furthermore, the answers to the question what else someone would do, showed that in total 72% of the sample would be ready to consult a psychiatrist or neurologist. Therefore, seeking professional help used to be and still is very important, however people prefer to talk to their acquaintances beforehand—something which has also been found in other studies (e.g., Ref. [11]).

This is of interest, as structural data illustrates that the number of mental health professionals in Austria has increased over the last 25 years [12, 13]. Systematically structured campaigns on mental health, in which Austria has a long tradition (e.g., the pilot project from “Open the doors” in 1996 [14, 15]), may have been limited in their numbers and effects (for an overview one may reference the 2017 contributions by Beldie and his coauthors [16]).

Our findings regarding the development of public attitudes towards those suffering from schizophrenia do only in part concur with the results of previous studies. As reported in the introduction, Schomerus et al.’s [1] meta-analysis of short and medium range trend studies had not shown changes in the public’s ascription of dangerousness to people with schizophrenia. However, two more recent long-term studies from Australia and the US found, similarly to what has been observed in Austria, a significant increase in perceived dangerousness. There is evidence that the belief in dangerousness is more likely to be due to media exposure than to personal experience of violent acts by mentally ill people [17, 18]. Although the last highly publicized case of murder committed by a mentally ill person occurred in Austria back in 2007 [19], more recent reports from other countries on violent acts by mentally ill people may have had a spill-over effect on the Austrian public’s perception. Particularly such incidents in neighboring Germany often find broad coverage in the Austrian media, as, for instance, the murder suicide of the co-pilot of a Germanwings plane in 2015 [20]. Moreover, media reports of multiple homicides by mentally ill people in the US have worldwide interest and may also have impacted the Austrian public’s belief in the dangerousness of people with mental illness. Jorm and Reavley [15] argue that in this way, “the US may be exporting stigma to the rest of the world” (p.215). The assumption of a link between media reporting and the ascription of violence is also supported by the result that parallel to the growing perception that people with schizophrenia tend to violence, the proportion of respondents claiming to have heard about schizophrenia in the context of reports on violent incidents has risen from merely 2.9% in 1998 to 24.9% in 2018 (change + 22; 95% CI 19.25; *p* < 0.001).

Although in other countries social acceptance of people with schizophrenia has not changed or has even changed for the worse, in Austria it has improved significantly. This is somewhat surprising concerning the afore mentioned increase in the ascription of violence. This indicates that both stigma domains may not be as closely linked, as results of some cross-sectional studies suggest [21]. Both, perceived violence and desire for social distance, may be driven by different factors that have developed differently over time. As previous studies have shown, personal contact with people with mental illness increases the willingness to engage in a social relationship with these people (e.g., [2]). In fact, familiarity of the Austrian public with people with schizophrenia has slightly grown over the past 20 years: although in 1998 9.4% of respondents reported to know someone with schizophrenia in their family or among their relatives and acquaintances, in 2018 the proportion has increased to 13.5% (change + 4; 95% CI 1.7; *p* = 0.005). Apart from that, a growing belief in the treatability of schizophrenia may also have contributed to the increase in social acceptance. In 1998 only 55.8% of respondents shared the view that the disorder is treatable, while 74.7% did so 20 years later (change + 19; 95% CI 15.23; *p* < 0.001).

The increase in social acceptance coincides with a decrease of causal attributions to genetic factors. This ties into a recent systematic review and a meta-analysis of population-based cross-sectional studies. There the authors concluded that a negative association between the ascription of biogenetic causes and social acceptance of people with schizophrenia exists [22, 23]. Trend studies point in the same direction, showing that a growing endorsement of biogenetic causes is accompanied by decreasing social acceptance [1]. However, our findings indicate that the inverse relationship between the two operates also the other way around, as in Austria the social acceptance increased significantly since 1998, while genetic explanations became less popular than before.

### Strengths and limitations of the study at hand

Spanning a time period of 20 years, our study is one of the longest vignette-based analyses of trends in public attitudes towards schizophrenia. Another strength is that in both surveys the same sampling procedure (quota sample), interview mode (face-to-face interview), and interview schedule was used, guaranteeing maximum comparability. On the other hand, the exclusive focus on attitudes may be seen a limitation since it allows predicting behavior with only limited accuracy. However, as Link et al. [24] have pointed out, apart from considering them as proxy for individual behaviors, public attitudes and beliefs can be conceptualized as a reflection of cultural conceptions of mental illness. Such conceptions form a cultural context that influences the way we think about mental illnesses and respond to those afflicted. To the extent that cultural conceptions are important, it becomes critical to understand them, which requires not only a single assessment but multiple assessments over long periods of time that allow to capture variations of these phenomena. Another limitation could be seen in the use of relative short vignettes, which can be understood as “proxies” for more detailed vignettes meeting the criteria of currently used diagnostic systems (e.g., [25]).

## Conclusion

The evolution of public beliefs about schizophrenia in Austria over the past 20 years shows a rather inconsistent pattern: While it has to be noted, that in some respects, the beliefs of the public have grown closer to the opinion of mental health professionals, in others they have not. Although there has been no increase in the readiness to seek professional help, the number of people suggesting that medication may help has grown. Similarly, attitudes towards people with schizophrenia did not develop uniformly: On the positive side, it has to be noted that the social acceptance has increased. However, at the same time, the ascription of violence also grew.

Furthermore, apart from similarities the findings show differences to time trend studies from other western countries. One can only speculate why this is the case. One explanation might offer the difference in the time period covered: While, for instance, the first survey was conducted in Austria in 1998, in Germany it had been already in 1990. It was, however, during the 1990s (the so-called “decade of the brain”) when in Germany a marked increase in the endorsement of biogenetic causes was observed, and correspondingly a steep decline in social acceptance of people with schizophrenia. Interestingly, during the following decade this development came to a halt and has even been reversed to some extent [26]. Thus, in the end, the discrepancies between both countries in the development of public beliefs and attitudes over time may have been less pronounced than one might assume at first glance.

A lesson to be learnt from our study is that trends in attitudes and beliefs observed in one country not necessarily can be generalized to other countries, even if they are culturally as close as Austria and Germany. This should be kept in mind when planning awareness-raising or stigma-reducing interventions. Stigma and the forms it may take depend strongly on the societal context that shapes it [27]. Our longitudinal case study in Austria highlights this.

Particularly disquieting is the growing ascription of violence to people with schizophrenia which is to be observed in Austria as well as in other countries. We are rather sceptic as regards the effect of public campaigns, as one carried out in Austria some 20 years ago and another one carried out only recently in Germany have failed in reducing the stereotype of dangerousness [17, 28]. In view of the afore mentioned link between media reporting and perceived violence it seems more promising to train journalists in more appropriate and less scandalizing reporting about violent incidences in which people with mental illness may be involved.

## Data Availability

The data are currently not publicly available; rights for the data collected during the remain with the funding agency, the involved researchers are allowed to use it for scientific publications.

## Code availability

SPSS Syntax is available; the data were processed via SPSS V26.

## References

[1] Schomerus G, Schwahn C, Holzinger A, Corrigan PW, Grabe HJ, Carta MC, Angermeyer MC (2012). Evolution of public attitudes about mental illness: a systematic review and meta-analysis. Acta Psychiatr Scand.

[2] Angermeyer MC, Matschinger H, Schomerus G (2013). Attitudes towards psychiatric treatment and people with mental illness: changes over two decades. Br J Psychiatry.

[3] Reavley NJ, Jorm AF (2014). The Australian public’s beliefs about the causes of schizophrenia: associated factors and change over 16 years. Psychiatry Res.

[4] Reavley NJ, Jorm AF (2012). Public recognition of mental disorders and beliefs about treatment: changes in Australia over 16 years. Br J Psychiatry.

[5] Reavley NJ, Jorm AF (2012). Stigmatizing attitudes towards people with mental disorders: changes in Australia over 8 years. Psychiatry Res.

[6] Pescosolido BA, Martin JK, Long SJ, Medina TR, Phelan JC, Link BG (2010). Evolving public views on the likelihood of violence from people with mental illness: Stigma and its consequences. Health Affairs.

[7] Grausgruber A, Katschnig H, Meise U, Schöny W (2001). Einstellungen der österreichischen Bevölkerung zu Schizophrenie [Attitudes of the Austrian public towards schizophrenia]. Neuropsychiatrie.

[8] Vannieuwenhuyze JT (2015). Mode effects on variances, covariances, standard deviations, and correlations. J Surv Stat Methodol.

[9] Prandner D, Röser A (2017). Questions of quality—is data quality still tied to survey mode? An Austrian case study dealing with attitudes concerning refugees. Medien J.

[10] Link BG, Cullen FT, Frank J, Wozniak JF (1987). The social rejection of former mental patients: understanding why labels matter. Am J Sociol.

[11] Angermeyer MC, Matschinger H, Riedel-Heller SG (2001). What to do about mental disorder—help-seeking recommendation of the lay public. Acta Psychiatry Scand.

[12] Sagerschnig S, Tanios A (2016) Psychotherapie, Klinische Psychologie, Gesundheitspsychologie. Statistik der Berufsgruppen 1991–2015 [Psychotherapy, clinical psychology, health psychology. Statistics for the professions between 1991 and 2015]. Gesundheit Österreich, Vienna. https://jasmin.goeg.at/413/1/Berufsgruppen_1991-2016_0-PDF_final.pdf. Accessed 10 Jun 2020

[13] Hauptverband der österreichischen Sozialversicherungsträger (2017) Vertragsärztinnen und -ärzte in Österreich. Bestandsaufnahme und Analyse. Wien https://www.sozialversicherung.at/cdscontent/load?contentid=10008.714994&version=1490770055. Accessed 20 Aug 2020

[14] Schöny, W (no Year) Open the doors – local action groups – Austria. https://www.openthedoors.com/english/01_05_01.html. Accessed 10 Jun 2020

[15] Schleicher F, Schöny W, Sartorius N, Schulze H (2005). AUSTRIA—reducing the stigma of mental illness. Reducing the stigma of mental illness: a report from a global association.

[16] Beldie A, Brain C, Figueira ML, Filipci I, Jakovljevic M, Jarema M, Gaebel W, Rössler W, Sartorius N (2017). Stigma in midsize European countries. The stigma of mental illness-end of the story?.

[17] Jorm AF, Reavley NJ (2014). Public belief that mentally ill people are violent: is the USA exporting stigma to the rest of the world?. Aust N Z J Psychiatry.

[18] Reavley NJ, Jorm AF, Morgan AJ (2016). Beliefs about dangerousness of people with mental health problems: the role of media reports and personal exposure to threat or harm. Soc Psychiatry Psychiatric Epidemiol.

[19] Grausgruber A, Schöny W, Grausgruber-Berner R, Koren G, Apor BF, Wancata J, Meise U (2009). „Schizophrenie hat viele Gesichter” – Evaluierung der österreichischen Anti-Stigma-Kampagne 2000–2002 [Schizophrenia has many faces—evaluating the Austrian Anti-Stigma-Campaign 2000–2002]. Psychiatr Prax.

[20] Schomerus G, Stolzenburg S, Angermeyer MC (2015). Impact of the Germanwings plane crash on mental illness stigma—results from two population surveys in Germany before and after the incident. World Psychiatry.

[21] Angermeyer MC, Matschinger H (2003). The stigma of mental illness: effects of labelling on public attitudes towards people with mental disorder. Acta Psychiatrica Scandinavia.

[22] Angermeyer MC, Holzinger A, Carta MG, Schomerus G (2011). Biogenetic explanations and public acceptance of mental illness. A systematic review of population studies. Br J Psychiatry.

[23] Kvaale EP, Gottdiener WH, Haslam N (2013). Biogenetic explanations and stigma: a meta-analytic review of associations among laypeople. Soc Sci Med.

[24] Link BG, Angermeyer MC, Phelan J, Thornicroft G, Szmukler G, Mueser KT, Drake RE (2011). Public attitudes towards people with mental illness. Oxford textbook of community mental health.

[25] Pescosolido BA, Medina TR, Martin JK (2013). The “Backbone” of stigma: identifying the global core of public prejudice associated. Am J Public Health.

[26] Schomerus G, Von der Auwera S, Matschinger H, Baumeister SE, Angermeyer MC (2015). Do attitudes towards persons with mental illness worsen during the course of life? An age-period-cohort analysis. Acta Psychiatr Scand.

[27] Angermeyer MC, Carta MG, Matschinger H, Millier A, Refaï T, Schomerus G, Toumi M (2016). Cultural differences in stigma surrounding schizophrenia: comparison between Central Europe and North Africa. Br J Psychiatry.

[28] Makowski AC, Mnich EE, Ludwig J, Daubmann A, Bock T, Lambert M, Härter M, Dirmaier J, Tlach L, Liebherz S, Von dem Knesebeck O (2016). Changes in beliefs and attitudes toward people with depression and schizophrenia—results of a public campaign in Germany. Psychiatry Res.

